# Testing the effectiveness of a mindfulness-based intervention to reduce emotional distress in outpatients with diabetes (DiaMind): design of a randomized controlled trial

**DOI:** 10.1186/1471-2458-11-131

**Published:** 2011-02-24

**Authors:** Jenny van Son, Ivan Nyklíček, Victor JM Pop, François Pouwer

**Affiliations:** 1 Center of Research on Psychology in Somatic diseases (CoRPS), Department of Medical Psychology and Neuropsychology, Tilburg University, Tilburg, The Netherlands

## Abstract

**Background:**

Approximately 20-40% of outpatients with diabetes experience elevated levels of emotional distress, varying from disease-specific distress to general symptoms of anxiety and depression. The patient's emotional well-being is related to other unfavorable outcomes, like reduced quality of life, sub-optimal self-care, impaired glycemic control, higher risk of complications, and increased mortality rates. The purpose of this study is to test the effectiveness of a new diabetes-specific, mindfulness-based psychological intervention. First, with regard to reducing emotional distress; second, with respect to improving quality of life, dispositional mindfulness, and self-esteem of patients with diabetes; third, with regard to self-care and clinical outcomes; finally, a potential effect modification by clinical and personality characteristics will be explored.

**Methods/Design:**

The Diabetes and Mindfulness study (DiaMind) is a randomized controlled trial. Patients with diabetes with low levels of emotional well-being will be recruited from outpatient diabetes clinics. Eligible patients will be randomized to an intervention group or a wait-list control group. The intervention group will receive the mindfulness program immediately, while the control group will receive the program eight months later. The primary outcome is emotional distress (anxiety, stress, depressive symptoms), for which data will be collected at baseline, four weeks, post intervention, and after six months follow-up. In addition, self-report data will be collected on quality of life, dispositional mindfulness, self-esteem, self-care, and personality, while complications and glycemic control will be assessed from medical files and blood pressure will be measured. Group differences will be analyzed with repeated measures analysis of covariance.

The study is supported by grants from the Dutch Diabetes Research Foundation and Tilburg University and has been approved by a medical ethics committee.

**Discussion:**

It is hypothesized that emotional well-being, quality of life, dispositional mindfulness, self-esteem, self-care, and blood pressure will improve significantly more in the mindfulness group compared to the control group. Results of this study can contribute to a better care for patients with diabetes with lowered levels of emotional well-being. It is expected that the first results will become available in 2012.

**Trial registration:**

Dutch Trial Register NTR2145.

## Background

It has been estimated that worldwide, approximately 285.000.000 persons have diabetes mellitus (DM) and this number is expected to rise to 439.000.000 by 2030 [1]. This sharp increase in prevalence is partly due to the ageing of the population and the increase in the number of people having overweight and who are physical inactive [2]. DM is a chronic disease characterized by high levels of blood glucose resulting from a deficit in the secretion of the hormone insulin (absolute insulin deficiency: Type 1 DM), or insufficient insulin action (insulin resistance) and/or a failure of the beta-cells to produce enough insulin, i.e. beta-cell dysfunction (Type 2 DM)[3]. Type 2 DM is the most frequent form, prevalent in approximately 90% of the cases [4]. For all patients with diabetes, appropriate glycemic control by means of adequate self-management is required in order to prevent or delay the development of diabetes complications: macrovascular complications (e.g. coronary artery disease) and microvascular complications (i.e. nephropathy, retinopathy, and neuropathy). This self-management consists for example of a healthy diet, physical exercise, frequent assessment of the blood glucose levels, and (in many cases) the use of medication to control the blood glucose (an oral hypoglycemic agent and/or insulin)[3].

A considerable proportion of the patients with diabetes (20-40%) experience emotional problems, which vary from disease-specific worries (such as fear of hypo's or worries about complications) to more general symptoms of distress, anxiety and depression [5-7]. These problems are not only unpleasant for the persons experiencing them, but studies show also that co-morbid emotional distress in patients with DM is associated with reduced quality of life [8], poor self-care behaviors [9], more negative appraisals of insulin therapy [10], reduced glycemic control [9] and subsequent adverse cardiovascular outcomes, and even mortality [8,11].

Despite the fact that emotional distress is so common in patients with diabetes, the attention for emotional problems in both practice and research is still limited. In practice, the recognition rates of emotional problems by clinicians are low and so are treatment rates [12]. Similarly, in the field of diabetes research, studies that test psychological interventions to improve the emotional well-being in patients are scarce. In addition, those studies that have been conducted often have important limitations, such as lack of controlled designs and small sample sizes [13]. However, it is true that the attention for emotional problems in DM patients is increasing since the last decade. In practice, screening for emotional problems is recommended in the latest versions of the standards of care of the International Diabetes Federation [14] and the American Diabetes Association [15]. In addition, in research, there are more and more studies examining the effectiveness of psychological interventions on clinical depression. Anxiety symptoms and diabetes-specific emotional distress have received, however, much less attention in diabetes research.

The results of well-designed studies that have been conducted to test psychological interventions in diabetes patients with emotional problems show that cognitive behavioral therapy is an effective treatment for major depression in diabetes patients [16] and a potentially effective treatment in reducing symptoms of anxiety, perceived stress, and diabetes-specific emotional distress [17,18]. Another promising psychological intervention that may help to reduce symptoms of depression, anxiety, and diabetes-specific emotional distress is based on the cultivation of mindfulness. Mindfulness can be defined as the self-regulation of one's attention focusing on direct experience, while adopting a curious, open, and accepting attitude towards these experiences, especially one's psychological processes, such as thoughts and feelings [19]. Mindfulness-based interventions can be considered promising as positive effects of mindfulness programs on emotional well-being and quality of life have been reported in diverse patients groups, like chronic pain patients and patients with cancer [20-22]. In addition, research has shown that the program is beneficial for patients with recurrent depression. In these patients, the intervention appeared to be superior to traditional cognitive behavioral therapy in the prevention of relapse of depression [23]. While in a recent meta-analysis, mindfulness-based interventions have been found to have medium to large effects regarding the reduction of symptoms of anxiety and depression [24], only two studies have focused on the effects of a mindfulness program on patients with diabetes. These studies showed that the interventions resulted in improved emotional well-being (Cohen's *d *= 0.86 for depressive symptoms and 0.43 for anxiety) [25], improved self-care behaviors (Cohen's *d *= 0.68)[26] and decreased HbA_1c _values (Cohen's *d *= 0.46) [25] (Cohen's *d *= 0.35)[26]. However, only one of the two studies was a randomized controlled trial, but this study did not include emotional well-being as an outcome measure [26]. Therefore, there is a clear need for randomized controlled studies in this area, examining the potential benefits of mindfulness interventions on emotional well-being in patients with DM.

In addition to the potential contribution of mindfulness to emotional well-being, mindfulness may also have a beneficial influence on self-management in DM patients. First, because mindfulness interventions enhance emotional well-being, and a better mood is associated with better self-care behavior, mindfulness is expected to enhance self-care behavior as well. Second, mindfulness improves one's ability to behave oneself constructively in harmony with his/her values, even during the experience of difficult thoughts and emotions [27]. Finally, because considerable focus is placed on bodily sensations during mindfulness interventions, mindfulness leads people to become more in touch with their body and its signals and needs [28], which is expected to lead to better self-care.

A positive effect of a mindfulness intervention may also be expected on blood pressure. It is known that episodic or chronic emotional distress can increase blood pressure [29]. This is unfavorable for patients with diabetes, as the prevalence of high blood pressure in people with diabetes is approximately twice as high compared to the general population [30] and both diabetes and high blood pressure are important risk factors for cardiovascular disease [30]. To date, only two studies have examined the influence of mindfulness on blood pressure, finding a reduction in mean arterial pressure in DM patients [25] and a reduction in resting systolic blood pressure in normotensive students [31].

If the mindfulness intervention appears to be effective in reducing emotional distress in DM patients, it is useful to know which patients will most likely benefit from the intervention. However, research into differences in effectiveness of the intervention between groups of patients with different characteristics (e.g. differences in personality and demographic and medical characteristics) is scarce.

### Objectives and hypotheses

Because of the paucity of studies on the effectiveness of psychological interventions for emotional problems in DM patients, especially regarding mindfulness based interventions, the purpose of this study is to test the effectiveness of a mindfulness-based psychological intervention. The primary outcome is emotional distress (symptoms of depression, anxiety, diabetes-specific stress, and general perceived stress). Secondary outcomes are quality of life, (diabetes-specific) mindfulness, and self-esteem, and our tertiary outcomes are self-management, glycemic control, and blood pressure. Finally, we will examine which patient characteristics (like the extent of complications and personality) predict which patients most likely benefit from the intervention.

With regard to our first outcome measure, we hypothesize that after an eight-week mindfulness-based training DM patients will experience significant greater reductions in emotional distress (diabetes-specific stress, anxiety, and depressive symptoms) compared to a wait-list control group. Concerning our secondary outcome measures, we hypothesize that the intervention will lead to greater improvements in psychological quality of life, dispositional (diabetes-specific) mindfulness, and self-esteem compared to a wait-list control group. In addition, we expect, consistent with two other studies [32,33], that changes in dispositional mindfulness will mediate the hypothesized effects of the intervention on above mentioned outcomes. Regarding our tertiary, more clinical, outcome measures, we hypothesize that the intervention will lead to better diabetes self-care, lower HbA_1c_, and lower BP in people with elevated BP. Finally, we are interested in personality and clinical factors that could possibly act as effect modifiers. We do not have clear expectations about the direction of the possible influence of these characteristics, being complications (yes/no), diabetes type (DM1 versus DM2), co-morbidity (yes/no), and personality.

## Methods/Design

### Study design

The Diabetes and Mindfulness (DiaMind) study will be conducted as a randomized controlled trial (RCT). Dutch speaking patients with DM with low levels of emotional well-being recruited from outpatient diabetes clinics will be randomly allocated to the intervention group or the wait-list (usual care) control group. The intervention group will receive the mindfulness program immediately, while the control group will receive the program eight months later (six months after the training of the intervention group).

### Eligibility

We will include adult men and women (aged 18 - 80 years) who were diagnosed with diabetes (type 1 or 2) and have poor emotional well-being as evidenced by a score of < 13 on the WHO-5 well-being Index [34]. Patients will be excluded when they have a recent history of severe psychopathology (i.e., psychosis, risk of suicide attempts); or alcohol/drugs abuse; have a severe physical co-morbidity (i.e., severe forms of cancer or heart failure); when they have insufficient reading and comprehension skills of the Dutch language; when they are already in an (extensive) psychological treatment which started within a period of 6 weeks before the start of the training; and when they already have meditation experience (with Vipassana, Zen, or Dzogchen).

### Recruitment and screening process and enrolment

The recruitment of patients will take place in outpatient diabetes clinics. At present, internists of the outpatient clinic of the Maxima Medical Center (Veldhoven and Eindhoven), the Catharina Hospital (Eindhoven) and the TweeSteden Hospital (Tilburg) have agreed to participate. Depending on the preferences of the diabetes teams in the participating hospitals, the screening will be conducted by the internists, diabetes nurses or by a researcher of our team. The screening tool will be the WHO-5 well-being Index [34]. This short (5-item) screening instrument, which assesses emotional well-being, is validated in the general population, but also in different patient samples, including patients with type 1 diabetes [35] and is recommended for use by the International Diabetes Federation (IDF) [14]. If patients have a low score on this scale (<13) they will receive an information letter about the training and study. One week later these patients will be telephoned and asked whether they are interested to participate in the project.

Eligible patients who are interested to participate will be invited for a short interview. This interview will take place in the hospital or, if logistically difficult for the patient, at their home. During this interview the in- and exclusion criteria will be checked once again, and expectations of the patient about the training will be checked and if necessary adjusted. Eligible patients will receive a written informed consent form during the interview that has to be signed and returned before the start of the training. Furthermore, at the end of the interview the blood pressure of the patients will be measured. If blood pressure is elevated (≥140/90 mmHg) the patient will be attached to an ambulatory blood pressure device which will measure blood pressure the following 24 hours. One day later the device will be brought back to the hospital by the patient or be picked up at the patient's home by a researcher.

### Randomization

After the inclusion of every 16 to 20 patients the participants will be randomized into two groups: the intervention group and the control group. The randomization will be done as follows. After the interview and upon receipt of a signed informed consent form, the patient will be assigned to a participant number. Subsequently the participant will receive the baseline questionnaire by mail or email. When the first author receives the baseline assessment, she passes on the corresponding participant number to the second author who has no further involvement in the practical recruitment, enrolment, and assessment of the patients. The second author will refer to the computer generated (through PASW Statisitics 17) random list (uneditable and concealed for others) prepared by a statistician with no involvement in the trial. The second author will inform the first author about the allocation by email and will archive the allocations in a secured document on his computer. The first author will document the allocation in the general inclusion database, which will be checked by the second author. The first author will inform the patients both by telephone and letter about their allocation. The above described procedure will eliminate experimenter bias in group assignment. The computer program the statistician will use utilizes a random number generator and will be programmed to insure equal numbers of subjects in each arm of the study within a block of 4 participants.

### Masking

The nature of this psychological intervention does not allow "masking" or blinding of patients, and trainers. However, the statistician who will be involved in data analyses will be blinded for treatment allocation. All the questionnaires and homework forms will only be marked with a participant number, which is unknown to the trainer and researcher.

### Intervention

#### Training

The protocolised mindfulness training is based on the Mindfulness-Based Stress Reduction and Mindfulness-Based Cognitive Therapy programs as described in Kabat-Zinn [28] and Segal et al. [36], consisting of eight weekly two-hour sessions. A few modifications have been made to the original protocol and the workbook in order to make the intervention suitable for patients with diabetes. For example, the trainers explain the potential associations between emotional problems and diabetes management and diabetes outcomes (e.g., the associations between emotional stress and eating behaviors will be discussed). Instead of the silent day that is part of the original program, a two-hour booster session has been added three months after the end of the intervention. At each session the participants will receive homework assignments that take about 30 minutes 5 days per week. All the sessions will be supervised by certified psychologists who have at least four years practical experience with mindfulness, and also completed a mindfulness instructors training of eight days in The Netherlands. The standard format of the sessions is as follows: the session starts with an exercise, which is followed by a discussion about the exercise and the homework exercises of the preceding week. Subsequently, a discussion about the theme of the session takes place, followed by the administration and discussion of one or two other mindfulness exercises. Finally, the homework assignments of the following week are discussed. All sessions end with a short meditation and/or a relevant poem or story.

##### Session 1

The first session of the course will consist of: 1) an invitation to all participants to introduce themselves sequentially, 2) a brief overview of the coming eight weeks, 3) a discussion about the relationship between diabetes, diabetes management, diabetes outcomes and emotional distress. After this, participants will be invited to slowly and mindfully eat a raisin with all their senses (the raisin exercise), which will be followed by a discussion about the fact that many people live their lives in a unmindful way, often do not pay much attention to what they are doing. Subsequently, the participants will be led through a body scan exercise of thirty-five minutes, during which the participants are encouraged to focus on the physical sensations in different body parts and to bring their attention back to that focus when they get distracted from it by thoughts or other momentary phenomena. At the end of the first session the participants will receive a CD containing several exercises and will be asked to practice the bodyscan on five occasions in the following week. In addition, they will be advised to perform one daily activity (e.g. to wash the dishes, to brush their teeth) in a mindful way and to eat one meal per day mindfully.

##### Session 2

At the beginning of the second session the participants will be guided again through the body scan, after which they will be invited to express and share their experiences with the mindfulness exercises and the homework of the preceding week. Subsequently there will be a discussion about coping with obstacles to doing the exercises (e.g., irritation, wandering mind) and attitudes that support the cultivation of mindfulness (e.g., not judging, letting go). After that, participants will be led through an exercise 'thoughts and feelings', which explains a basic component of cognitive behavioral therapy: our emotions are not caused by events but by our perception of events. Subsequently, the participants will practice 'sitting meditation', with breathing as the primary object of attention. Home practice for the following week will include awareness of pleasant events, a new routine daily activity, the bodyscan, and brief sitting meditation.

##### Session 3

The third session will start with a short 'Seeing and/or hearing exercise', in which participants are instructed to exercise non-judgmental seeing or hearing for a couple of minutes. This exercise will be followed by a sitting meditation with focus on breathing and bodily sensations. After the homework discussion, there will be a talk by the trainer about attention to breathing, followed by the practice and discussion of the '3-minute breathing space'. This meditation has three phases: attention to the experiences in the moment, attention to the breathing, attention to the body. Subsequently, an exercise in mindful body movements will be introduced and discussed. Exercises to be practiced during the following week will include sitting meditation, bodyscan or body movement exercises, 3-minute breathing space, new mindful daily activity, and awareness of unpleasant events.

##### Session 4

The fourth sessions will start with a sitting meditation with attention to the breathing, the body, sounds, and thoughts (so called 'sitting meditation with four focuses'). Subsequently, there will be a discussion about the stress response and common reactions of individuals to difficult situations, and alternative attitudes and reactions will be discussed. Then a Dutch documentary about MBSR (entitled 'Aandachttraining') will be shown to the participants. At the end of the session an exercise in mindful walking will be introduced and practiced. The homework assignments for this fourth session will consist of practicing sitting meditation, the bodyscan or mindful body movements, 3-minute breathing space (also during unpleasant events), to fill in the questionnaire 'automatic thoughts' (optional), and to read an inspiring book about mindfulness (optional).

##### Session 5

At the beginning of the fifth session the participants will be guided through the sitting meditation with four focuses (see Session 4). A poem will be read and the psychologist will discuss the theme of the fifth session: 'Acknowledging and accepting the reality of the present situation as it is.' Subsequently, the second series of mindful body movements will be introduced. The recommended home work will consist of sitting meditation, 3-minute breathing space, 3-minute breathing space during unpleasant events, and a new daily activity performed mindfully.

##### Session 6

The sixth session will begin with the 3-minute breathing space. The homework assignments will be discussed mindfully in pairs. An exercise called 'mood, thoughts, and different view points' will be discussed plenary, as well as the theme of the sixth session: 'The content of our thoughts is (often) not factual'. Subsequently, the participants will be guided through four linked up meditation exercises, with a total duration of one hour. For the following week participants will be encouraged to choose a combination of meditations that fits their needs and personal preferences. In addition, the home practice includes the 3-minute breathing space (also during unpleasant events), a new mindful daily activity, and having a mindful conversation while paying attention to one's automatic patterns (optional).

##### Session 7

The seventh sessions will start with the meditation exercise with four focuses and open awareness (to whatever enters consciousness from moment to moment). The theme of the session, 'What is the best way to care for oneself', will be discussed, followed by an exercise during which participants explore daily activities that are pleasant versus unpleasant and learn to plan sufficient pleasant activities. The 3-minute breathing space is performed, followed by a loving-kindness meditation during which one practices kindness towards oneself and others. The home practice includes a meditation combination of one's choice, 3-minute breathing space (also during unpleasant events), a new daily activity performed mindfully, and filling in a form including warning signals for emotional distress.

##### Session 8

The session will begin with a body-scan. The theme of the last session will be discussed: 'Using what you have learned'. Subsequently the psychologist will guide the participants through a 3-minute breathing space and will read a story called 'Five chapters', which is about the steps one can take to overcome our pitfalls in the practice of mindfulness. Finally, the whole training will be evaluated with the participants. For example the following questions will be asked: did the training meet their expectations, do participants have a sense of 'personal growth', do they feel that they have expanded their coping skills and do they want to continue practicing mindfulness. In addition, resources for continued practice will be given (e.g. titles of books and addresses of local meditation centers).

### Evaluation

After completing the training the patients will be invited for a short interview, in which the patient's perceptions regarding the quality of the intervention will be evaluated and the procedure with the ambulant blood pressure measurement will be repeated.

### Data collection

Table 1 shows the assessment instruments and data collection time points. Assessments will take place before the start of the intervention, at 4 weeks, at 8 weeks (= post intervention), at 12 weeks and at 34 weeks (6 months post intervention). The control group will receive another four measures, namely at 38 weeks (4 weeks after start of control group intervention), at 42 weeks (post control group intervention), at 46 weeks, and at 62 weeks (6 months post control group intervention). Process variables (e.g., attendance, adherence, and drop-out) will be collected continuously. The patients who have access to the internet and email will receive the questionnaire via the internet. If this is not the case, they will receive a paper version of the questionnaire along with a stamped addressed envelope.

**Table 1:** Measurements and time points

Concept	Questionnaire		Measurement time points							
		
		T1	T2	T3	T4	T5	T6	T7	T8	T9
Perceived stress	PSS (14)*	**x**	**x**	**x**		**x**	x	x		x

Anxiety and depressive symptoms	HADS (14)	**x**	**x**	**x**		**x**	x	x		x

Mood	POMS (32)	**x**	**x**	**x**		**x**	x	x		x

Diabetes related problems	PAID (20)	**x**	**x**	**x**		**x**	x	x		x

Quality of life	SF12 (12)+3 items WHOQOL	**x**		**x**		**x**		x		x

Self-care behavior	ADSCI (11)	**x**		**x**		**x**		x		x

Extraversion	EPQ-E (12)	**x**								

Type D Personality	DS14 (14)	**x**		**x**		**x**		x		x

Mindfulness	FFMQ(31)	**x**		**x**		**x**		x		x

Diabetes related mindfulness	AADQ (11)	**x**		**x**		**x**		x		x

Self-esteem	RSES (10)	**x**		**x**		**x**		x		x

Patient's background (demographics, clinical, psychological)	- (21)	**x**								

**Clinical measurements**	**Measurement**									

Glycemic control (from hospital database)	hbA1c	**x**			**x**	**x**			x	x

Blood pressure	Amb.24 h BP	**x**		**x**						

T1 - baseline; T2 - after 4 weeks of intervention; T3 - after 8 weeks (post intervention); T4 - after 12 weeks (4 weeks post intervention); T5 - after 8 months (6 months post intervention = start training for control group). T6 thru T9 only for control group: repetition of T2 thru T5. PSS - Perceived Stress Scale; HADS - Hospital Anxiety and Depression Scale; POMS - Profile of Mood States; PAID - Problem Areas in Diabetes Survey; SF-12 - Short Form Health Survey; 3 items WHOQOL-bref^9^; ADSCI - Amsterdam Diabetes Self Care Inventory; EPQ-E - Eysenck Personality Questionnaire subscale Extraversion; DS14 - Type D personality scale; FFMQ - Five Factor Mindfulness Questionnaire; AADQ - Acceptation and Action Diabetes Questionnaire; RSES - Rosenberg Self-esteem Scale. HbA1c = hemoglobin A1c (the amount of glycated hemoglobin in blood). Amb. 24 h BP = Ambulatory 24 hour Blood Pressure monitoring.

*= number of items in questionnaire. Total number of items = 205.

### Outcome parameters

#### 1. Primary outcome measures

##### Stress, anxiety and depressive symptoms

We will include the Hospital Anxiety and Depression Scale (HADS) to measure symptoms of anxiety (HADS-A: e.g., "Worrying thoughts go through my mind") and depression (HADS-D: e.g., "I feel as I am slowed down") in DM patients [37]. The self-report scale consists of 14 items, answered on a 4-point Likert scale, with the HADS-A and HADS-D both comprising 7 items (*0-3*). The score range for the anxiety and the depressive symptoms subscales is 0 to 21. The HADS has been validated in the general population and in somatic and psychiatric patients [38,39]. It has been shown to be a valid and reliable instrument with Cronbach's α ranging from 0.67 to 0.93 for the two subscales [37-39].

The Dutch version of the Perceived Stress Scale (PSS) will be included to measure general perceived stress (the degree to which situations in one's life are appraised as stressful) (e.g. "In the last month, how often have you felt nervous and stressed?"). It has been shown to be a reliable measure with Cronbach's α ranging from 0.84 to 0.86 [40]. The present version consists of 10 items which are answered on a five-point Likert scale, ranging from 'never' to 'very often' *(0-4) *[41].

The short Dutch version of the Profile of Mood States (POMS) [42] will be used to assess transient, fluctuating mood states. The scale consists of 32 adjectives about positive and negative mood states which have to be rated on a five-point Likert scale *(0 = not at all, 4 = very much) *based on how well each item describes one's mood during the last couple of weeks. For the interpretation of the results the items are divided into five subscales: Tension-anxiety (6 items); Depression-dejection (8 items); Anger-hostility (7 items); Vigor-activity (5 items); and Fatigue-inertia (6 items). The scale has sufficient consistency reliability and construct validity, with Cronbach's α's of the five subscales varying between 0.82 and 0.91 [42,43].

##### Diabetes specific emotional problems

We will include the Dutch version of the self-report questionnaire Problem Areas in Diabetes Survey (PAID), which consists of 20 statements about common negative feelings related to living with diabetes (e.g., "Feeling depressed when you think about living with diabetes", "feeling discouraged with your diabetes regimen") [44]. The items are rated on a six-point Likert scale *(1 = not a problem, 6 = a serious problem)*. PAID scores are transformed to a 0-100 scale, to facilitate interpretation [45]. A higher score indicates more emotional distress, with a cut-off score of 40 indicating seriously elevated emotional distress [46]. Factor analysis revealed that the factor structure can be represented by a one factor model (PAID total: emotional adjustment) and a four factor model (subscales: emotional problems, treatment problems, food problems, and lack of social support) [45]. We will examine the results with both models. The survey has proven to be a reliable measure, with a Cronbach's α of 0.95 for the one factor model. For the four factor model the Cronbach's α's are in range of 0.93 (emotional problems), 0.74-0.76 (treatment problems), 0.70-0.74 (food problems), and 0.69-0.72 (lack of social support) [45]. In addition, research in Dutch and American patients found support for convergent and discriminative validity of the PAID [45,47].

#### 2. Secondary outcome measures

##### Quality of life

We will include the Dutch version of the Short-Form Health Survey (SF-12) to measure health related quality of life. The self-report scale consists of 12 items that are grouped into two component summary scores: a physical (PCS) and a mental component score (MCS). Both component scores are measured on a scale from 0 to 100, with a high score indicating good health related quality of life. The SF12 has proven to be a valid and reliable measurement [48].

In addition, we will use three items of the WHOQOL-BREF [49] to measure satisfaction with oneself and life in general: "How much do you enjoy life?"; "To what extent do you feel your life to be meaningful?"; "How satisfied are you with yourself?". The questions will be analyzed separately at item level if Cronbach's α will show to be below 0.65.

##### Mindfulness

To measure mindfulness we will include the Dutch version of the Five Facet Mindfulness Questionnaire (FFMQ). FFMQ is based on a factor analytic study of five independently developed mindfulness measurement scales [50]. The factors we will use are: *Observing*, which refers to noticing or attending to internal and external experiences, such as thoughts, sensations, emotions, sounds, sights, and smells; *Acting with awareness*, which includes attending to one's activities in the present moment; *Non-judging of inner experience*, which refers to taking a non-evaluative attitude toward thoughts and feelings; and *Non-reactivity to inner experience*, which includes allowing thoughts and feelings to come and go, without getting caught up in or carried away by them [51]. The factor *Describing *will be omitted, because describing one's emotions and feelings is not a primary focus of MBSR/MBCT and we do not want to burden our patients unnecessarily. Each factor consists of 7 or 8 items, which will be answered on a five-point Likert scale (*1 = never or very rarely true, 5 = very often or always true*). The four facets demonstrated adequate to good internal consistency (alphas ranging from 0.75 to 0.87) [50].

Changes in diabetes-specific mindfulness and acceptance processes will be assessed by the Acceptance and Action Diabetes Questionnaire (AADQ) [26]. This self-report questionnaire consists of 11-items that are answered on a 7-point Likert scale. It measures acceptance of diabetes-related feelings and thoughts and the extent to which they interfere with valued action (e.g., "I avoid thinking about what diabetes can do to me") [26]. For this measure, a Cronbach's α of 0.94 has been reported [26].

##### Self-esteem

Self-esteem will be measured by the Dutch version of the Rosenberg Self-Esteem Scale (RSES), wherein self-esteem is defined as a person's overall evaluation of his or her worthiness as a human being [52]. The questionnaire consists of 10 items answered on a four-point Likert scale, ranging from 'I totally disagree' to 'I totally agree'. A sample item is "I take a positive attitude toward myself". A higher score reflects a higher global self-esteem. The Cronbach's alpha was .86, indicating a high internal consistency [52].

#### 3. Tertiary outcome measures

##### Self-care behavior

Self-care behavior (or self-management) will be assessed with the Amsterdam Diabetes Self Care Inventory (ADSCI). The questionnaire has 11 items, which will be examined separately. Each item is divided into three or four subquestions. Higher scores indicate higher levels of self-care. The questionnaire is developed by colleagues of the VU University Medical Center in Amsterdam and used in previous trials [53,54]. The psychometric properties of the ADSCI have not yet been published.

##### Glycemic control

To measure glycemic control we will consult the three-monthly standard measurements of HbA1c of the hospital, which are documented in the hospital's patient database. The percentage HbA1c is a function of the glucose concentration to which the red blood cells are exposed and gives an indication of the average blood glucose concentration of the preceding six to eight weeks [3].

##### Blood pressure

The patients' blood pressure will be measured by an ambulant device, called the Mobil-O-Graph, which is based on the oscillometric method and which has been shown to provide reliable and valid assessments [55]. The patients, who have elevated high blood pressure (≥ 140 mmHg systolic or 90 mmHg diastolic of the mean values of 3 measurements) during the interview, will wear the meter 24 hours following the interview. The ambulatory measurements will occur two times per hour. To keep the burden for the patients as low as possible, we decided to measure the blood pressure only during the daytime. The device will be attached to the patient by the researcher who does the interviews (JvS).

#### 1. Additional outcome measures

##### Personality

To measure personality dimensions we will use the subscale Extraversion (EPQ-E) of the shortened revised Eysenck Personality Questionnaire (EPQ-RSS) and the DS-14 to measure Type D personality. Persons with a Type D personality tend to experience high levels of emotional distress, but do generally not express these emotions. For the EPQ-E, the subscale Extraversion reflects sociability, assertiveness, and the tendency to experience positive emotions. The subscale consists of 12 dichotomous (yes/no) items [56]. The Committee on Test Affairs Netherlands rates the EPQ to be a reliable measure [57].

The 14-item Type D scale (DS14) measures both negative affectivity (NA) (e.g. "I often feel unhappy"; 7 items) and social inhibition (SI) (e.g. "I am a closed kind of person"; 7 items) [58]. Items are answered on a five-point Likert scale ranging from 'false' to 'true' *(0-4)*, with the score range for the NA and SI subscales being 0 to 28 [58]. We will use the standardized cut-off ≥ 10 on both subscales to identify Type D caseness [58,59]. The DS14 has been shown to be a valid and reliable instrument, with Cronbach's α of 0.88 and 0.86 and a 3-month test-retest reliability of r = 0.72 and 0.82 for the NA and SI subscales, respectively [58]. Furthermore, a recent study showed that Type D personality is a stable construct over an 18-month period and is not confounded by disease severity and measures of anxiety and depression [60,61]. Results from a study by Denollet et al [62] suggest that particularly the interaction between NA and SI is predictive of adverse health outcomes, more than the single traits [62].

##### Demographic and clinical variables

Demographic variables will be collected by means of a questionnaire, which will be completed by the patients during the baseline assessment. Data will be collected regarding the participant's age, marital status, education, and job status. In addition, the baseline questionnaire will be used to gather information on history of emotional problems and use of psychotropic medication, level of meditation experience, sleep quantity and quality, participant's perceived importance of adequate blood glucose regulation, and amount of participant's effort to manage their blood glucose. Furthermore, the baseline questionnaire will be used to assess clinical data, such as length and weight of participants, last HbA1c, number of severe hypoglycemic episodes and/or hospitalizations because of a diabetic coma during the past year, existing diabetes complications, co-morbidities, and smoking and drinking behavior. The clinical variables (which are available) will also be retrieved from the hospitals' patient information database.

#### 1. Additional process measures

During the intervention process data will be collected, like drop-out, patients' attendance of the sessions, patients' adherence to the home work assignments, and trainers' adherence to the protocol. The adherence to the protocol will be determined each session by the trainers by means of the completion of a checklist including all components of each session. The components are divided into important and less important, facilitating decision making when time constraints are present.

### Statistical analyses

#### Sample size and power calculation

The total number of patients that have to be included and complete the study is 160, with 80 patients in each arm, taking into account a medium effect size, a power of 0.80, an alpha of 0.05, patient attrition, and subgroup analyses involving two equally sized groups (e.g. yes vs. no complications). It is expected to reach this number of participants during the course of the study.

#### Planned analyses

Repeated measures analysis of variance ((M)ANCOVA) will be used to test the hypotheses concerning the differences between groups on the dependent variables over time. In these tests age, sex, and co-morbidity will be included as covariates, in addition to those other potentially confounding variables that will show an at least marginally significant (p < .10) difference between groups. The analyses concerning the subgroup effects will be conducted on the sample as a whole, whereby possible moderating variables, like complications and personality will be included in the analyses as between-subjects factors. All analyses will be based on the intention-to-treat approach.

### Ethical principles

The study protocol has been approved by the medical ethical committee of the St. Elisabeth Hospital in Tilburg, The Netherlands (P0948).

## Discussion

The present article provides an outline of the background and design of the Diabetes and Mindfulness (DiaMind) study. This study's objective is to test an intervention based on mindfulness training supplemented with elements from cognitive therapy that aims to improve emotional well-being of distressed patients with diabetes. For this purpose, an intervention group will be compared with a wait-list control group with treatment as usual.

Research on psychological interventions that aim to reduce emotional problems in DM patients is important, as emotional problems are associated with a higher risk of impaired quality of life [8], less optimal self-care [9], incident complications, and mortality [8,11]. However, to date there are few methodologically well-designed studies on the effectiveness of psychological interventions for people with diabetes and emotional complaints. In addition, the majority of these studies have focused on improving self-management instead of emotional well-being. Moreover, the few randomized studies that have examined the effectiveness of interventions in reducing emotional problems in people with diabetes often focused on depression. For this reason, this study is clearly of added value in the research field of diabetes and emotional problems, as we have taken a broader perspective and focused on emotional distress (depression, anxiety, and general distress).

The study design has a number of important strengths. First, the present study has more power to generate knowledge on the effectiveness of a mindfulness-based intervention for DM patients with emotional problems than former studies examining mindfulness in DM patients. As mentioned before, only two studies have been done on this subject, of which one lacked a control group and one did not include emotional well-being as an outcome [25,26]. Second, an additional strength of this study is the inclusion of both psychological and clinical measures, while most randomized controlled trials examining mindfulness interventions include psychological measures only. Finally, another strong aspect of this study is the examination of factors that potentially moderate the effectiveness of the mindfulness intervention, including clinical (e.g. complications) and psychological (e.g. personality) variables. Results may facilitate more effective allocation of patients to treatments.

The recruitment has already started in the Catharina Hospital in Eindhoven in spring 2010 and in the Maxima Medical Center in autumn 2010. At present, 969 individuals have been screened for emotional problems. Of those, 811 did not qualify: 658 had a score above 12 on the WHO-5 screening instrument, and 153 were ineligible (no mastery of the Dutch language, psychiatric disorder, or severe physical condition). Of the eligible patients, 120 declined participation (no perceived need or motivation for the training or an incompatible working schedule). At present, 42 patients are interested to participate, of whom 16 have been enrolled so far.

The recruitment of a sufficient number of patients will be a challenge. For instance, the mindfulness intervention requires participants to commit to attending eight two-hour group sessions and to practice exercises at home for five days per week. For people who have busy lives this sometimes is a challenge to comply with, which may make them decline participation or drop out of the study.

Concerning the benefits of the present study, the results will provide valuable information regarding the effectiveness of the mindfulness intervention for DM patients in improving emotional well-being. We will also gain insight in the pros and cons of a possible implementation of the intervention in usual medical care of DM patients. By doing this, we aim to find ways that can contribute to increased emotional well-being and quality of life in patients with diabetes mellitus.

In conclusion, considering the prospected increase of the prevalence of diabetes, the levels of emotional distress in this patient group, and the incompleteness of the current literature on effective psychological interventions for the distressed patients, well-designed trials on psychological interventions are a welcome addition in the diabetes research. In the present randomized controlled trial the effectiveness of a psychological intervention will be examined in which the cultivation of mindfulness (the direction of attention to one's experiences in the present moment in an open and accepting manner) plays a central role. This intervention may be an effective alternative to traditional cognitive behavioral therapy in improving emotional well-being in DM patients. The first results of the study will become available in 2012.

## Competing interests

The authors declare that they have no competing interests.

## Authors' contributions

All authors contributed to the design of the study. IN is the principal investigator of the study. IN and FP coordinate and supervise JvS (a PhD-student). JvS drafted the manuscript. All authors contacted professionals (i.e. internal medicine doctors) involved in the recruitment of patients. JvS is responsible for the logistics of the study, and takes care of the recruitment of participants and data collection. IN, FP and JvS prepared the patients' work book. JvS prepared the detailed protocol for the trainers of the intervention. All authors provided comments, read and approved the various versions including the final version of the manuscript.

## Pre-publication history

The pre-publication history for this paper can be accessed here:

http://www.biomedcentral.com/1471-2458/11/131/prepub
